# A luminescent Cd(ii) coordination polymer as a multi-responsive fluorescent sensor for Zn^2+^, Fe^3+^ and Cr_2_O_7_^2−^ in water with fluorescence enhancement or quenching†

**DOI:** 10.1039/d0ra10203b

**Published:** 2021-03-17

**Authors:** Liangjuan Liu, Yungen Ran, Jianlong Du, Zhichao Wang, Mei Liu, Yajuan Mu

**Affiliations:** College of Traditional Chinese Medicine, Hebei University Baoding 071000 P. R. China muyjhbu@hbu.edu.cn; College of Life Science, Hebei University Baoding 071000 P. R. China; College of Chemistry & Environmental Science, Hebei University Baoding 071000 P. R. China

## Abstract

A luminescent Cd(ii) coordination polymer, namely {[Cd(btic)(phen)]·0.5H_2_O}_*n*_ (CP-1) (H_2_btic = 5-(2-benzothiazolyl)isophthalic acid, phen = 1,10-phenanthroline), was constructed through the mixed-ligand method under solvothermal conditions. CP-1 manifests a chain structure decorated with uncoordinated Lewis basic N and S donors. CP-1 exhibits high sensing towards Zn^2+^, Fe^3+^ and Cr_2_O_7_^2−^ ions with fluorescence enhancement or quenching. CP-1 exhibited a fluorescence enhancement for Zn^2+^ ions through weak binding to S and N atoms, and a fluorescence quenching for Fe^3+^ and Cr_2_O_7_^2−^ ions by an energy transfer process. The binding constants were calculated as 1.812 × 10^4^ mol^−1^ for Zn^2+^, 4.959 × 10^4^ mol^−1^ for Fe^3+^ and 1.793 × 10^4^ mol^−1^ for Cr_2_O_7_^2−^. This study shows CP-1 as a rare multi-responsive sensor material for the efficient detection of Zn^2+^, Fe^3+^ and Cr_2_O_7_^2−^ ions.

## Abstract 2

A luminescent Cd(ii) coordination polymer can act as a multi-responsive sensor for efficiently detecting Zn^2+^, Fe^3+^ and Cr_2_O_7_^2−^ ions.

## Graphical abstract

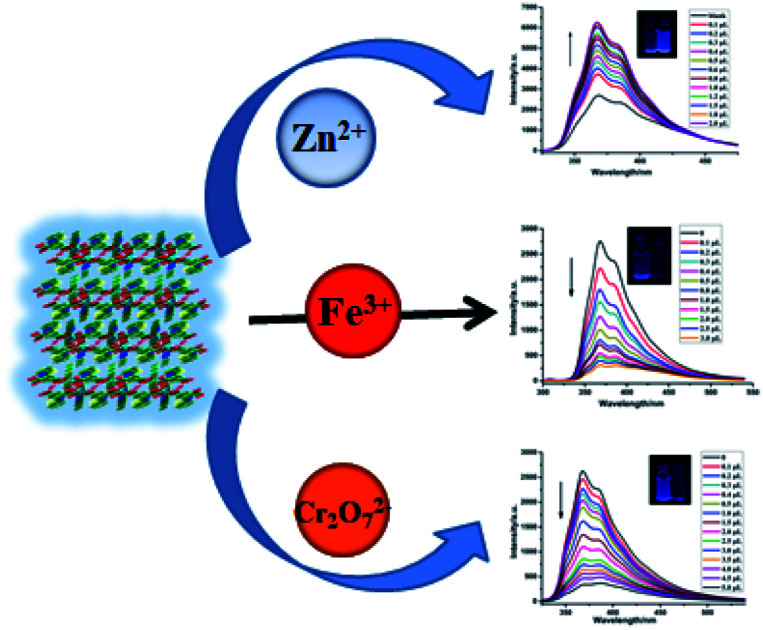

## Introduction

Zn^2+^ and Fe^3+^ are essential metal ions, which are involved in numerous biological processes in human body.^1–6^ The abnormal levels of Zn^2+^ and Fe^3+^ can cause numerous adverse health effects. Hence, efficient methods for the detection of Zn^2+^ and Fe^3+^ are highly desirable. On the other hand, Cr_2_O_7_^2−^ is a type of environmentally non-biodegradable pollutant, which can cause accumulation in living organisms and lead to the visceral damage and water-borne diseases because of its potent mutagenesis and carcinogenesis.^7–9^ Therefore, methods for the efficient detection of Cr_2_O_7_^2−^ are urgent to be explored. Currently, several traditional methods have been developed for the determination of Zn^2+^, Fe^3+^ and Cr_2_O_7_^2−^, such as atomic absorption spectrophotometry,^10–12^ electrochemical methods,^13–15^ and inductively coupled plasma mass spectrometry.^16–18^ However, these methods are usually expensive, time-consuming, or require complicated sample preparation processes. Therefore, simple and efficient detection methods for such analytes are urgently required.

Coordination polymers (CPs) are coordination compounds with infinite structures (1, 2 or 3 dimensions), which are constructed from metal ions and ligands *via* coordination bonds.^19^ CPs as a type of promising functional materials have received considerable attention because of their intriguing structural features as well as applications in luminescence sensing, magnetic property, gas storage, drug delivery, and so on.^20–28^ Particularly, CPs as fluorescent sensors have drew more concern owing to their superiority in long-term stability, efficiency, and operability. Simultaneously, much effort has been made to synthesize fluorescent CP materials for detecting different types of target analytes, such as metal ions,^29,30^ anions,^31,32^ and small molecules.^33,34^ However, many of these specific target analytes are detected in organic solvents, such as ethanol,^35,36^ DMF,^37–39^ CH_3_CN,^40^ DMA,^41^ which are not beneficial for the practical applications of fluorescent CPs. Therefore, detecting analytes in water is still an important challenge in biological and environmental sciences.

Organic ligands are crucial in the syntheses of fluorescent CPs. Aromatic or conjugated π moieties within organic ligands can endow the CPs with excellent optical properties, which can improve the efficient recognition for the target analytes.^42–44^ Moreover, the Lewis basic sites in fluorescent CPs can interact with certain metal cations, which can enhance the selective sensing capacity towards metal cations.^45^ Based on the above considerations, we adopted a multidentate ligand 5-(2-benzothiazolyl)isophthalic acid (H_2_btic) as a main ligand, which contains two aromatic rings. Such ligand was accompanied by an auxiliary ligand with excellent optical properties 1,10-phenanthroline (phen) to fabricate a luminescent CP, namely {[Cd(btic)(phen)]·0.5H_2_O}_*n*_ (CP-1). CP-1 magnified a chain structure decorated with uncoordinated Lewis basic N and S donors. The luminescence sensing behavior of CP-1 was studied in water solution. The as-synthesized CP-1 showed a striking sensing capability towards Zn^2+^, Fe^3+^ and Cr_2_O_7_^2−^ ions with fluorescence enhancement or quenching. In addition, the mechanism of luminescence enhancement or quenching has also been discussed.

## Experimental section

### Materials and instruments

All commercially available chemicals were used as received. Elemental analyses were conducted using a Flash EA 1112 elemental analyzer. IR data were obtained using a BRUKER TENSOR 27 spectrophotometer. PXRD patterns were obtained on a Bruker AXS D8Advance. Thermogravimetric analyses were measured on a NetzschSTA449C thermal analyzer under air atmosphere at a ramp rate of 10 °C min^−1^. The luminescence properties were studied using a Hitachi F-7000 fluorescence spectrophotometer. XPS data were obtained on a Thermo ESCALAB 250 X-ray photoelectron spectrometer.

### Synthesis of the ligand H_2_btic

Ligand H_2_btic was synthesized according to the reported procedure.^46^ The detailed synthetic method is described in the ESI (Section 1†).

### Synthesis of {[Cd(btic)(phen)]·0.5H_2_O}_*n*_ (CP-1)

A mixture of Cd(NO_3_)_2_·4H_2_O (0.1 mmol), H_2_btic (0.1 mmol), and phen (0.05 mmol) in 5 mL of the component solvent (DEF : H_2_O = 4 : 1) (DEF = *N*,*N*′-diethylformamide) was put into a glass vessel, which was heated at 80 °C until the colorless crystals appeared. Yield: 68% (based on Cd). Anal. calcd for C_54_H_32_N_6_O_9_S_2_Cd_2_ (%): C, 54.15; H, 2.69; N, 7.02; S, 5.35. Found: C, 53.68; H, 2.51; N, 7.10; S, 5.19. IR (cm^−1^, KBr): 3422(s), 3131(w), 3060(w), 2970(w), 2926(w), 1610(s), 1551(s), 1513(m), 1433(s), 1364(s), 1310(w), 1249(w), 1139(w), 1104(m), 1031(w), 934(w), 887(w), 777(s), 722(s), 686(w), 638(m).

### Fluorescence sensing experiments

1 mg of CP-1 was placed separately into 2 mL aqueous solutions containing numerous analytes (10^−3^ M). After an ultrasonic treatment for 15 min, the photoluminescence responses were recorded with excitation at 278 nm. The fluorescence titrations were conducted through adding analytes into 2 mL suspension of CP-1.

### X-ray crystallography

X-ray diffraction data were collected on a Bruker SMART APEX-II CCD diffractometer. The crystal structure was solved and refined using the SHELX-2014 software.^47^ Hydrogen atoms were added geometrically. Crystallographic parameters for CP-1 are shown in Table 1. Table 2 shows the main bond lengths and bond angles.

**Table 1:** Crystal data and structure refinement details for CP-1

Compound	1
Formula	C_54_H_32_N_6_O_9_S_2_Cd_2_
fw	1195.76
Crystal system	Monoclinic
Space group	*P*2/*c*
*a*/Å	10.1136(10)
*b*/Å	9.7173(10)
*c*/Å	24.005(3)
*α*/deg	90
*β*/deg	92.928(4)
*γ*/deg	90
*V*/Å^3^	2356.1(4)
*Z*	2
2*θ*_max_ (deg)	55.082
*D*_c_/g cm^−3^	1.686
Reflns collected/unique	48 969/5434
*R*(int)	0.0699
Abs coeff/mm^−1^	1.059
*F*(000)	1192
GOF	1.054
*R*_1_ [*I* > 2*σ*(*I*)]^a^	0.0415
w*R*_2_ (all data)^b^	0.0791
Largest diff. Peak and hole	0.64 and −0.70 e Å^−3^

a *R*_1_ = ∑‖*F*_o_| − |*F*_c_‖/∑|*F*_o_|.

b w*R*_2_ = [∑*w*(*F*_o_^2^ − *F*_c_^2^)^2^/∑*w*(*F*_o_^2^)^2^]^1/2^.

**Table 2:** Selected bond lengths (Å) and bond angles (deg) for CP-1^a^

CP-1			
Cd(1)–O(3)#1	2.236(2)	Cd(1)–N(2)	2.314(3)
Cd(1)–O(1)#2	2.353(2)	Cd(1)–N(1)	2.371(3)
Cd(1)–O(2)#2	2.402(3)	Cd(1)–O(1)	2.522(2)
Cd(1)–O(4)#1	2.528(3)	O(3)#1–Cd(1)–N(2)	135.13(9)
O(3)#1–Cd(1)–O(1)#2	101.27(10)	N(2)–Cd(1)–O(1)#2	86.18(9)
O(3)#1–Cd(1)–N(1)	101.03(11)	N(2)–Cd(1)–N(1)	71.08(10)
O(1)#2–Cd(1)–N(1)	155.64(9)	O(3)#1–Cd(1)–O(2)	82.98(9)
N(2)–Cd(1)–O(2)	137.08(9)	O(1)#2–Cd(1)–O(2)	107.77(10)
N(1)–Cd(1)–O(2)	84.58(11)	O(3)#1–Cd(1)–O(1)	129.17(8)
N(2)–Cd(1)–O(1)	95.62(8)	O(1)#2–Cd(1)–O(1)	75.24(9)
N(1)–Cd(1)–O(1)	97.87(9)	O(2)–Cd(1)–O(1)	52.41(8)
O(3)#1–Cd(1)–O(4)#1	54.00(8)	N(2)–Cd(1)–O(4)#1	81.42(8)
O(1)#2–Cd(1)–O(4)#1	95.36(9)	N(1)–Cd(1)–O(4)#1	89.88(10)
O(2)–Cd(1)–O(4)#1	134.66(8)	O(1)–Cd(1)–O(4)#1	170.36(9)

a Symmetry transformations used to generate equivalent atoms in CP (1): #1 −1 + *x*, *y*, *z*; #2 1 − *x*, *y*, 3/2 − *z*.

## Results and discussion

### Crystal structure of CP-1

**Fig. 1:**
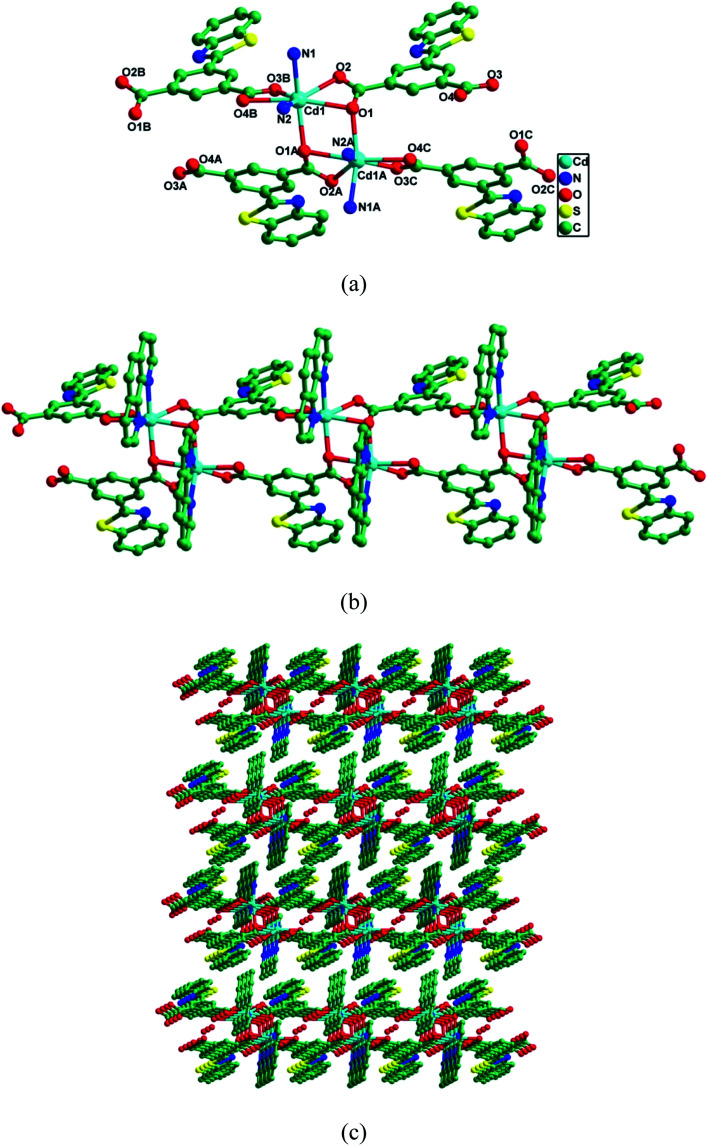
(a) Coordination environment around the Cd(ii) center in CP-1. Hydrogen atoms and solvent molecules are omitted for clarity. Symmetry codes: *A* = 1 − *x*, *y*, 1.5 − *z*; *B* = −1 + *x*, *y*, *z*; *C* = 2 − *x*, *y*, 1.5 − *z*. (b) The chain structure of CP-1. (c) The 3D supramolecular structure of CP-1.

CP-1 crystallizes in the monoclinic space group *P*2/*c*. As depicted in Fig. 1a, the asymmetric unit contained one Cd(ii) ion, one btic^2−^ and one phen, and half lattice H_2_O. Each Cd(ii) ion adopted a pentagonal-bipyramidal environment finished by five O atoms of three btic^2−^ and two N atoms of one phen. The Cd–O/N bond lengths varied from 2.236(2)–2.528(3) Å, and O/N–Cd–O/N bond angles were 52.41(8)–170.36(9)°, which coincided with those of the reported Cd(ii) CPs.^48–50^ Different coordination modes were shown by the two carboxylate groups of btic^2−^, namely μ_2_-η^1^:η^2^ and μ_1_-η^1^:η^1^ modes. Then, Cd(ii) ions were connected by carboxylate groups from btic^2−^ ligands to produce a Cd(btic) chain (Fig. 1b). The Cd(btic) chain was made up of dinuclear units Cd_2_(COO)_2_. The phen ligands took bidentate chelating fashion coordinating to Cd(ii) ions to satisfy the coordination demands of Cd(ii) ions in the assembly process. As shown in Fig. 1c, these chains in an offset way were stacked into a 3D supramolecule *via* weak van der Waals interactions. Further, the π–π interactions in each chain between pyridine rings (N2–C16–C17–C18–C19–C20) of phen ligands stabilized the structure. In CP-1, the N and S donors in ligand btic^2−^ were not involved in the coordination to Cd(ii) ions. Therefore, the uncoordinated N and S could function as Lewis bases to recognize numerous analytes.

### PXRD and thermogravimetric analysis

To check the phase purity of CP-1, its PXRD was performed (Fig. S1†). The peak positions of the as-synthesized sample were consistent with those of the simulated ones. The crystals of CP-1 were stable in air. Moreover, it did not dissolve in water or common organic solvents. To check the chemical stability of CP-1, each finely ground powder of CP-1 was immersed in methanol, ethanol, DMF, H_2_O and THF solvents for 24 h. Then, the PXRD of each sample was analyzed (Fig. S2†). The unchanged PXRD patterns revealed that the crystallinity of CP-1 retained after the solvent treatment, indicating the high chemical stability of CP-1. Thermogravimetric analysis was carried out to understand the thermal stability of CP-1 (Fig. S3†). The TGA curve of CP-1 exhibited an initial weight loss of 2.21% from 108 to 193 °C because of the release of H_2_O molecules (calcd: 1.51%). The further weight loss from 349–640 °C was ascribed to the disintegration of the structure, leaving CdO as the residue (found, 22.13%; calcd, 21.73%).

### Photoluminescence properties

At ambient temperature, the solid-state luminescences of H_2_btic, phen, and CP-1 were measured (Fig. S4†). When excited at 278 nm, H_2_btic had an emission maximum at 433 nm as well as phen showed a main peak at 382 nm with two shoulder peaks at 365 nm and 403 nm. The excitation of CP-1 at 278 nm led to a dominant peak at 388 nm with two shoulder peaks at 375 nm and 409 nm, which probably originated from intraligand transitions because similar emission was observed for ligand phen. CP-1 showed a small redshift compared to that of phen, which was probably because of the coordination effect of ligands to Cd(ii) ions. Further, a stronger emission band in CP-1 was probably assigned to the enhanced rigidity of the ligand that diminished a radiationless decay through the coordination to metal centers.^51–53^

### Sensing of metal ions

**Fig. 2:**
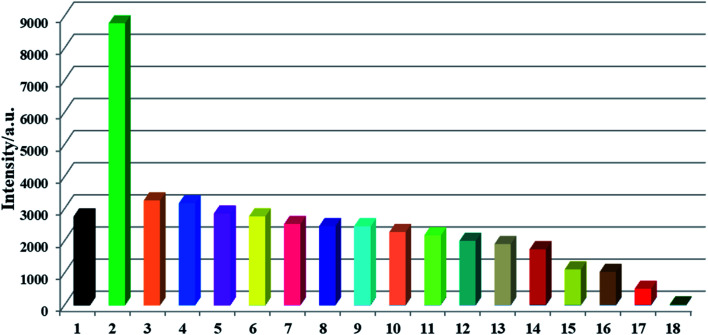
Luminescence intensity histograms of CP-1 (1 mg) dispersed in aqueous solutions of numerous metal ions (2 mL, 10^−3^ M) excited at 278 nm (1) blank, (2) Zn^2+^, (3) Ca^2+^, (4) Ba^2+^, (5) Mg^2+^, (6) K^+^, (7) Na^+^, (8) Cd^2+^, (9) Mn^2+^, (10) Al^3+^, (11) Ag^+^, (12) Cr^3+^, (13) Ni^2+^, (14) Co^2+^, (15) Pb^2+^, (16) Hg^2+^, (17) Cu^2+^, (18) Fe^3+^.

The Lewis basic N and S active sites, good chemical stability and strong luminescence for CP-1 made it a potential candidate as a fluorescent sensor. Thus, we explored the application of CP-1 in detecting metal ions. 1 mg powder of CP-1 was placed separately in 2 mL 10^−3^ M M^*x*+^ aqueous solutions (M^*x*+^ = Na^+^, K^+^, Mg^2+^, Ca^2+^, Cr^3+^, Ag^+^, Zn^2+^, Hg^2+^, Pb^2+^, Ni^2+^, Co^2+^, Cd^2+^, Ba^2+^, Al^3+^, Cu^2+^, Mn^2+^, and Fe^3+^). Then, luminescence responses toward different metal ions were examined. As shown in Fig. 2, these metal ions exhibited different impacts on the fluorescence intensities of CP-1. Notably, Zn^2+^ ions enhanced the emission intensity by 2.17-fold, Fe^3+^ ions almost completely quenched the luminescence, while other metal ions had little or moderate quenching effects on the emission, indicating that CP-1 could act as a sensor material with a great response for Zn^2+^ and Fe^3+^ ions.

**Fig. 3:**
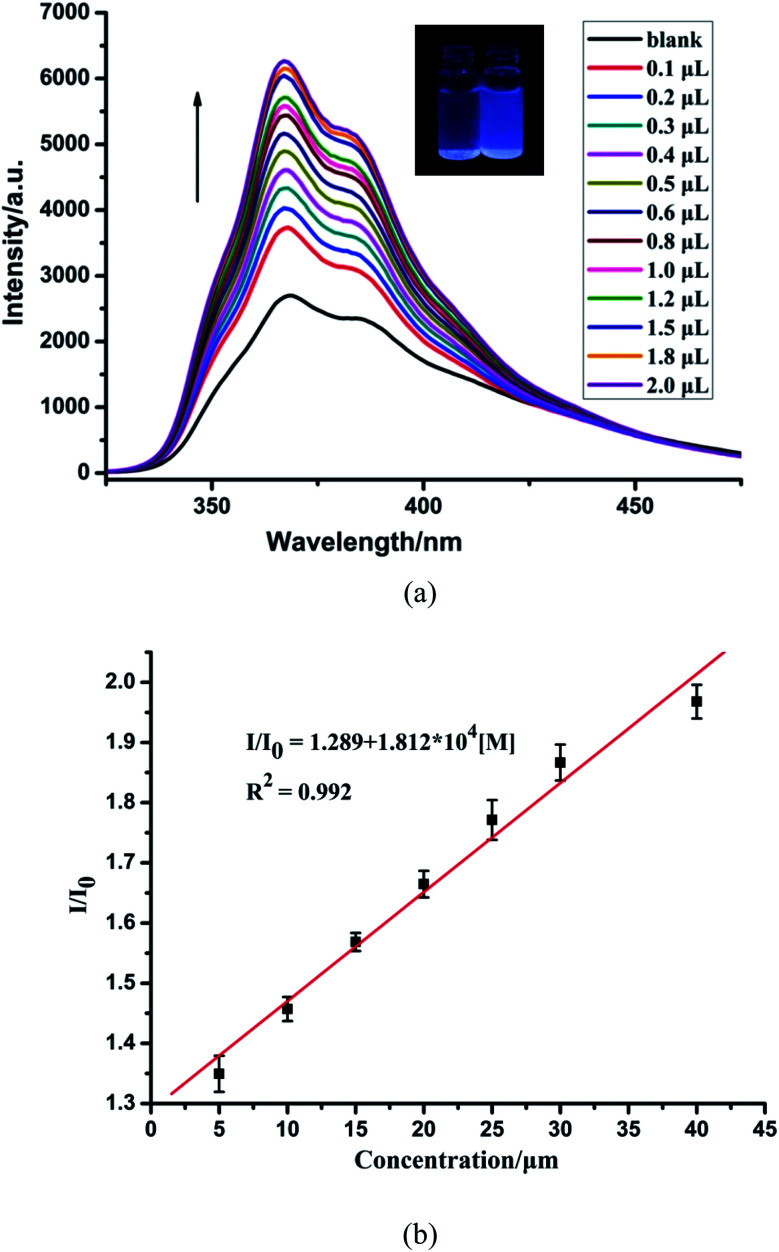
(a) Fluorescence spectra of CP-1 dispersed in an aqueous suspension upon the incremental addition of Zn^2+^ ions. (b) The linear correlation for the plot of *I*/*I*_0_*vs.* the concentration of Zn^2+^.

Moreover, to check the sensing sensitivity of CP-1 towards Zn^2+^, the following experiment was carried out. The sample of CP-1 was dispersed in water (0.5 mg mL^−1^), and executed with an ultrasonic treatment to obtain a suspension. Then, different volumes of Zn^2+^ ions (0.1 M) were added to the above suspension, and the emission spectra were determined (Fig. 3a). With incremental addition of Zn^2+^, the emission intensities gradually increased. The association constant was calculated to be 1.812 × 10^4^ mol^−1^ based on the fitted linear equation *I*/*I*_0_ = 1.289 + 1.812 × 10^4^ [M], where *I*_0_ and *I* are fluorescence intensities before and after analyte incorporation, respectively, and [M] represents the concentration of the analyte (Fig. 3b). The limit of detection (LOD) for Zn^2+^ was 4.172 × 10^−4^ M. To the best of our knowledge, the reported sensors for Zn^2+^ are mostly based on organic molecules or composite materials,^54–56^ and CP-based fluorescent sensors for detecting Zn^2+^ ions are very rare.

**Fig. 4:**
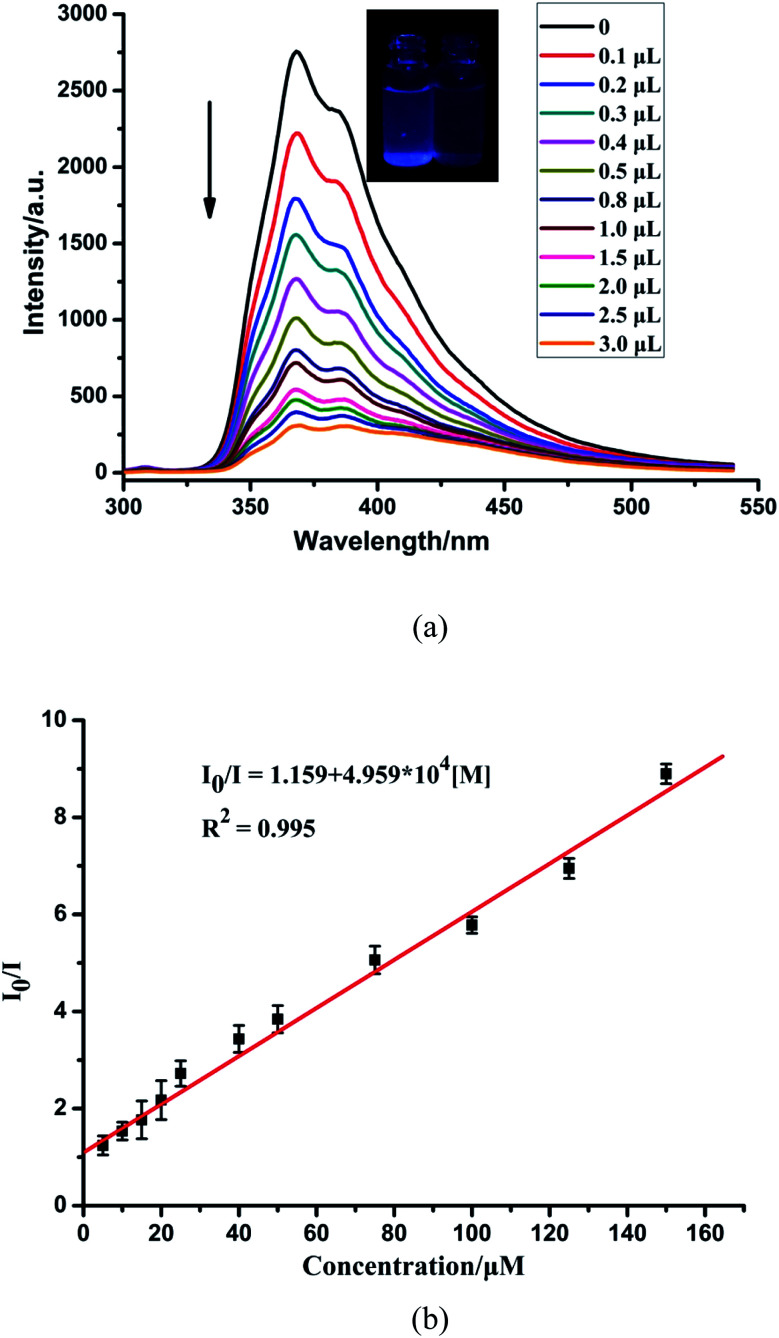
(a) Fluorescence spectra of CP-1 dispersed in an aqueous suspension upon the incremental addition of Fe^3+^ ions. (b) The linear correlation for the plot of *I*_0_/*I vs.* the concentration of Fe^3+^.

The sensing sensitivities of CP-1 for Fe^3+^ ions were also checked with the same method as in case of Zn^2+^. The increasing concentration of Fe^3+^ resulted in a gradual decrease in the fluorescent intensity of CP-1 (Fig. 4a). The quenching efficiency was estimated through the equation: *I*_0_/*I* = 1.159 + 4.959 × 10^4^ [M] (Fig. 4b). The quenching coefficient was calculated as 4.959 × 10^4^ mol^−1^ for Fe^3+^. The LOD was 1.524 × 10^−4^ M for Fe^3+^.

### Sensing of anions

**Fig. 5:**
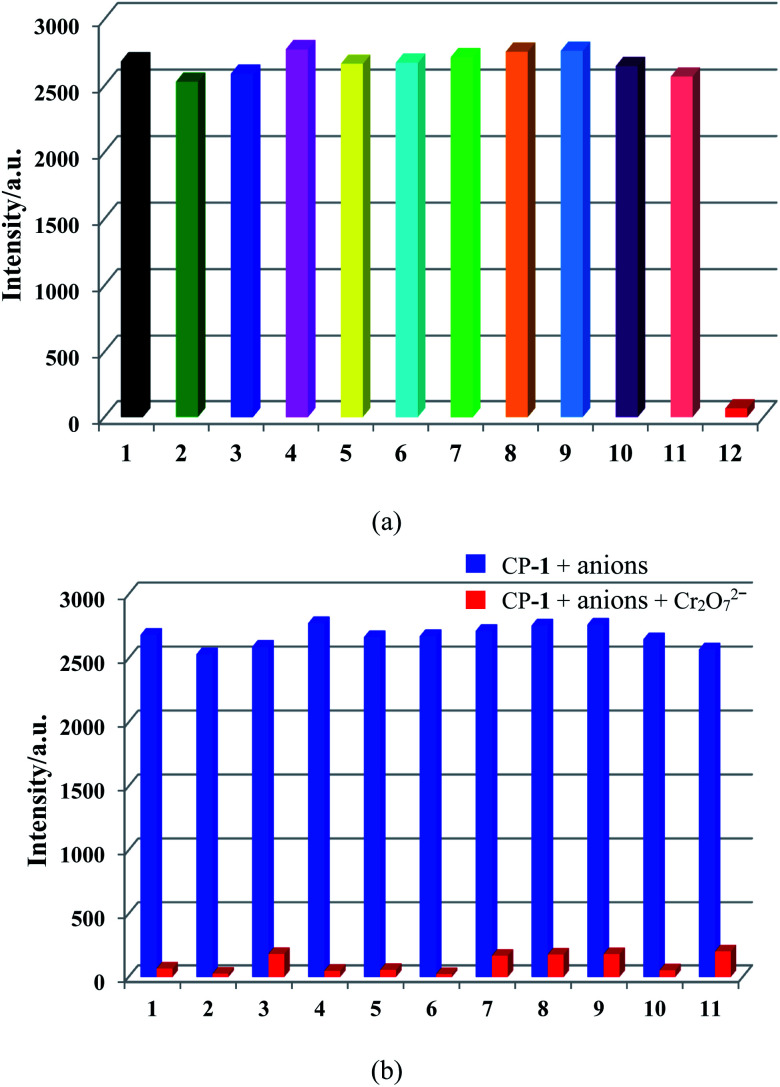
Luminescence intensity histograms of CP-1 (1 mg) dispersed into various anion aqueous solutions (2 mL, 10^−3^ M) excited at 278 nm (a) and subsequent addition of Cr_2_O_7_^2−^ (b). (1) Blank, (2) F^−^, (3) Cl^−^, (4) Br^−^, (5) I^−^, (6) CH_3_COO^−^, (7) SO_4_^2−^, (8) H_2_PO_4_^−^, (9) ClO_4_^−^, (10) SO_3_^2−^, (11) SCN^−^, (12) Cr_2_O_7_^2−^ (b).

Simultaneously, to check the effects of anions on the luminescence intensity of CP-1, numerous anions (F^−^, Cl^−^, Br^−^, I^−^, NO_3_^−^, CH_3_COO^−^, SCN^−^, ClO_4_^−^, H_2_PO_4_^−^, SO_3_^2−^, SO_4_^2−^, and Cr_2_O_7_^2−^) were selected. 1 mg of CP-1 was dispersed in aqueous solutions containing above anions (2 mL, 10^−3^ M). Obviously, Cr_2_O_7_^2−^ ions quenched the emission of CP-1, while other anions almost led to a little change in the fluorescence intensity of CP-1, indicating the potential detection of CP-1 towards Cr_2_O_7_^2−^ (Fig. 5a). Then, the sensing selectivity towards Cr_2_O_7_^2−^ was explored through competition experiments. Upon adding Cr_2_O_7_^2−^ into the mixture of CP-1 and each of other anions, the emission for each case was quenched greatly, showing the high selectivity of CP-1 towards Cr_2_O_7_^2−^ even in the presence of other interfering anions (Fig. 5b).

**Fig. 6:**
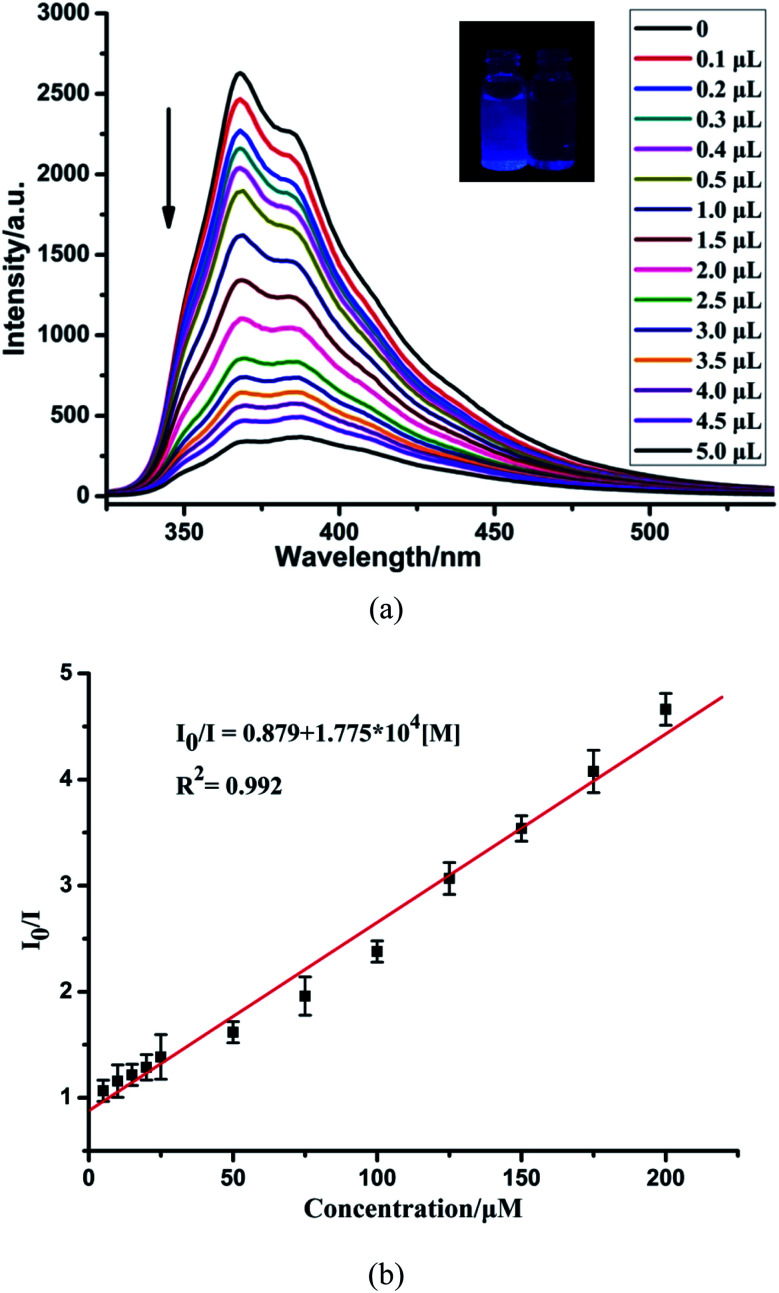
(a) Fluorescence spectra of CP-1 dispersed in an aqueous suspension upon the incremental addition of Cr_2_O_7_^2−^ ions. (b) The linear correlation for the plot of *I*_0_/*I vs.* the concentration of Cr_2_O_7_^2−^.

To further investigate the sensing capability of CP-1 towards Cr_2_O_7_^2−^, fluorescence titrations were conducted through the addition of different volumes of the Cr_2_O_7_^2−^ (0.1 M) aqueous solution to the suspension of CP-1. The emission intensity of CP-1 was gradually quenched with incrementally adding Cr_2_O_7_^2−^ (Fig. 6a). The quenching equation could be written as *I*_0_/*I* = 0.879 + 1.775 × 10^4^ [M] (Fig. 6b). The quenching coefficient was calculated as 1.793 × 10^4^ M^−1^. The LOD was calculated to be 4.21 × 10^−4^ M.

### The mechanism of luminescence sensing

To elucidate the reasons for the enhancement caused by Zn^2+^, and the quenching caused by Fe^3+^ and Cr_2_O_7_^2−^, the sensing mechanisms were also investigated. First, the samples were immersed in the aqueous solutions with numerous analytes for 16 h. Then, the PXRD patterns were recorded to examine the stability of the structure. As confirmed by the PXRD patterns (Fig. S5†), the whole skeleton of CP-1 remained unchanged after immersed in the Zn^2+^ aqueous solution. In the structure of CP-1, there were uncoordinated Lewis basic N and S atoms from ligand H_2_btic. Therefore, the hypothesis was that the uncoordinated N and S atoms possibly coordinated to Zn^2+^ ions, which increased the delocalization of CP-1 and improved the energy-transfer efficiency from ligand to metal ions, and finally enhanced the whole fluorescence intensity. To further prove the hypothesis, the XPS of N 1s and S 2p were carried out on CP-1 and Zn^2+^@CP-1 (Fig. S6†). The N 1s peak at 398.65 eV in CP-1 was shifted to 398.75 eV, while the S 2p peak at 163.45 eV in CP-1 shifted to 163.3 eV after Zn^2+^ incorporation, indicating the formation of weak bonds between S/N and Zn^2+^. The formation of weak bonds caused the efficient energy transfer between ligand and metal ions, which further led to the fluorescence enhancement.

After immersed in Fe^3+^ and Cr_2_O_7_^2−^ aqueous solution, the PXRD patterns showed that the skeleton of CP-1 also did not change, indicating that the fluorescence quenching was unrelated to the structure of CP-1 (Fig. S7 and S8†). Then, the energy transfer mechanism was checked through the measurement of UV-vis spectra of Fe^3+^ and Cr_2_O_7_^2−^, and the emission spectrum of CP-1, which depended upon the degree of overlap of the two kinds of spectra. As shown in Fig. S9 and S10,† the spectrum of CP-1 partly overlapped with absorption spectra of Fe^3+^ and Cr_2_O_7_^2−^, while other metal ions or anions displayed no spectral overlap. Therefore, the energy transfer mechanism could account for the luminescence quenching effects induced by Fe^3+^ and Cr_2_O_7_^2−^ ions.

## Conclusions

In summary, we synthesized a Cd(ii) coordination polymer with a chain structure (CP-1) based on the mixed-ligand method under solvothermal conditions. The excellent characteristics of high chemical stability, intense fluorescence, and the uncoordinated Lewis basic N and S atoms in the structure of CP-1 made it a rare multi-responsive fluorescence sensor to detect Zn^2+^, Fe^3+^ and Cr_2_O_7_^2−^ with fluorescence enhancement or quenching. This study also demonstrates that the introduction of functional groups or atoms in the ligands can endow the CPs with expected properties.

## Conflicts of interest

There are no conflicts to declare.

## Supplementary Material

RA-011-D0RA10203B-s001

RA-011-D0RA10203B-s002
