# Supplementary material for: A luminescent Cd(ii) coordination polymer as a multi-responsive fluorescent sensor for Zn2+, Fe3+ and Cr2O72− in water with fluorescence enhancement or quenching
Source: RSC Adv. 2021 Mar 17;11(19):11266–72. doi: 10.1039/d0ra10203b (PMC8695774; doi:10.1039/d0ra10203b)
Supplement: RA-011-D0RA10203B-s001 [file RA-011-D0RA10203B-s001.pdf]

Electronic Supplementary Material (ESI) for RSC Advances.
This journal is © The Royal Society of Chemistry 2021

A luminescent Cd(II) coordination polymer as a multi-responsive fluorescent sensor for Zn^{2+}, Fe^{3+} and $Cr_2O_7^{2-}$ in water with fluorescence enhancement or quenching

Liangjuan Liu,[a] Yungen Ran,[b] Jianlong Du,[c] Zhichao Wang,[a] Mei Liu,[a] Yajuan Mu,*[a]

[a] *College of Traditional Chinese Medicine, Hebei University, Baoding, 071000, P. R. China*

[b] *College of Life Science, Hebei University, Baoding, 071000, P. R. China*

[c] *College of Chemistry & Environmental Science, Hebei University, Baoding, 071000, P. R. China*

* Corresponding author. *E-mail: muyjhbu@hbu.edu.cn*

Section 1. Synthesis of the ligand H_2btic. **Page 3**

Figure S1. Experimental (red) and simulated (black) PXRD patterns of CP-**1**. **Page 4**

Figure S2. PXRD patterns of the as-synthesized CP-**1**, the simulated one and the samples after immersion in different solvents. **Page 5**

Figure S3. TGA curve of CP-**1**. **Page 6**

Figure S4. Solid-state photoluminescent spectra of free ligands and CP-**1** at room temperature. **Page 7**

Figure S5. PXRD patterns of the simulated pattern (Black), experimental pattern (Red), and the sample of CP-**1** after Zn^{2+} test (Blue). **Page 8**

Figure S6. N 1s and S 2p XPS spectra of CP-**1** (black) and Zn^{2+}-incorporated CP-**1** (red) activated by 0.001 mol/L aqueous solution of Zn^{2+}. **Page 9**

Figure S7. PXRD patterns of the simulated pattern (Black), experimental pattern (Red), and the sample of CP-**1** after Fe^{3+} test (Blue). **Page 10**

Figure S8. PXRD patterns of the simulated pattern (Black), experimental pattern (Red), and the sample of CP-**1** after $Cr_2O_7^{2-}$ test (Blue). **Page 11**

Figure S9. UV-vis absorption spectra of varied metal ions and the emission spectra of CP-**1** in water. **Page 12**

Figure S10. UV-vis absorption spectra of varied anions and the emission spectra of CP-**1** in water. **Page 13**

Section 1. Synthesis of the ligand H_2btic.

3,5-dimethylbenzoic acid (7.50 g, 0.05 mol), 2-aminothiophenol (6.76 g, 0.05 mol) and polyphosphoric acid (PPA) (150 mL) was added to a 500mL round bottomed flask. Under the nitrogen atmosphere, the mixture was heated at 200°C for 24 h. The reaction solution was poured into water. The crude precipitate was filtered off and washed with water with 0.1 M Na_2CO_3, then washed with water, finally recrystallized from ethanol. The obtained product (11.96 g, 0.05 mol) and $KMnO_4$ (7.90 g, 0.5 mol) were dissolved in 350 mL of a mixed solvent of pyridine/water (1/1). The reaction was heated at 90°C for 24 h. The solution was filtered to eliminate the by-product MnO_2. The remaining clear solution was acidized to pH = 3 with 2 M HCl. The resulting precipitate was dissolved in 1 M NaOH and reprecipitated by acidification to pH = 3 with 1 M HCl. The precipitate was filtered off, washed with water and dried to afford H_2btic as white solid. The reaction route is shown in Scheme S1. ^{1}H NMR (400MHz, DMSO-d_6): δ 13.698 (s, 2H), 8.784 (s, 2H), 8.601 (s, 1H), 8.227 (d, 1H), 8.195 (d, 1H), 7.620(m, 2H).

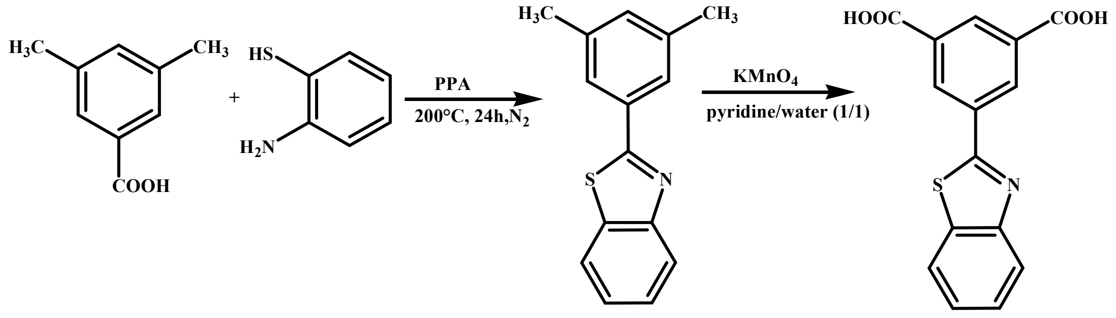

Scheme S1. The synthesis of ligand H_2btic.

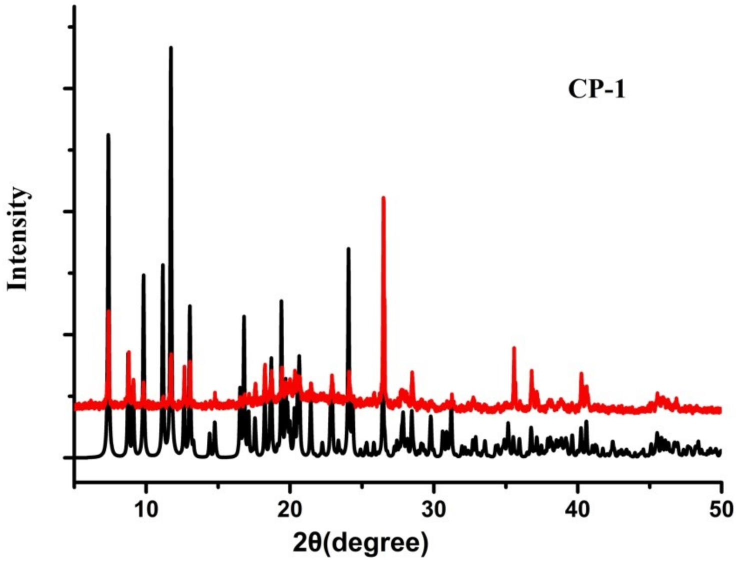

Figure S1. Experimental (red) and simulated (black) PXRD patterns of CP-**1**.

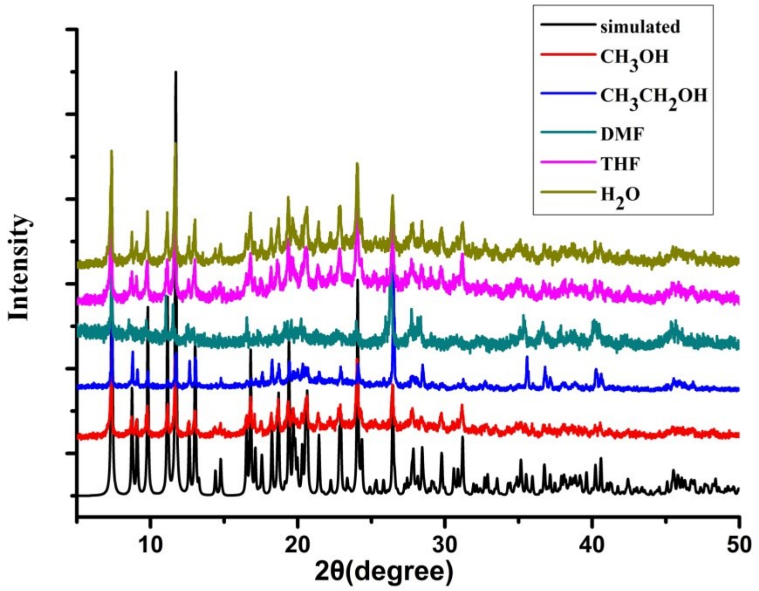

Figure S2. PXRD patterns of the as-synthesized CP-**1**, the simulated one and the samples after immersion in different solvents.

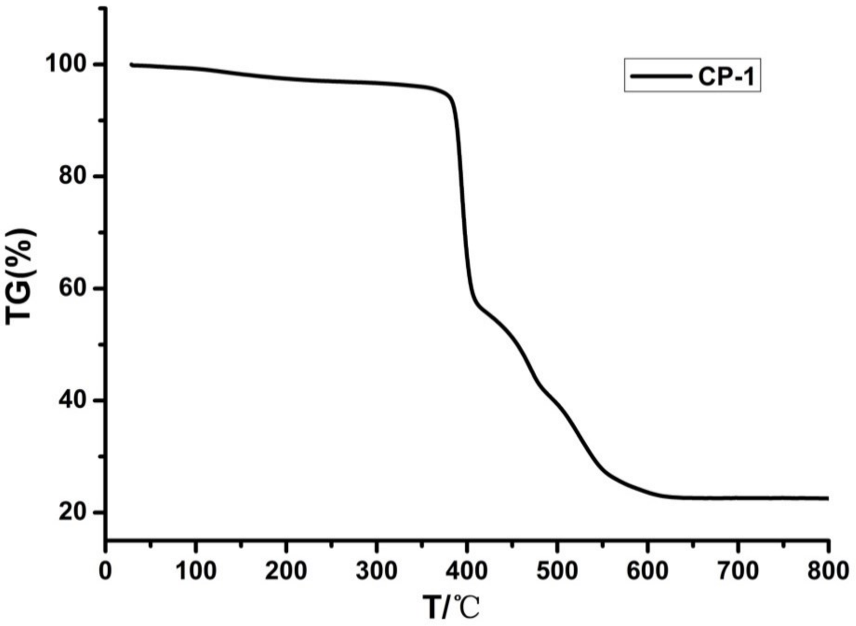

Figure S3. TGA curve of CP-**1.**

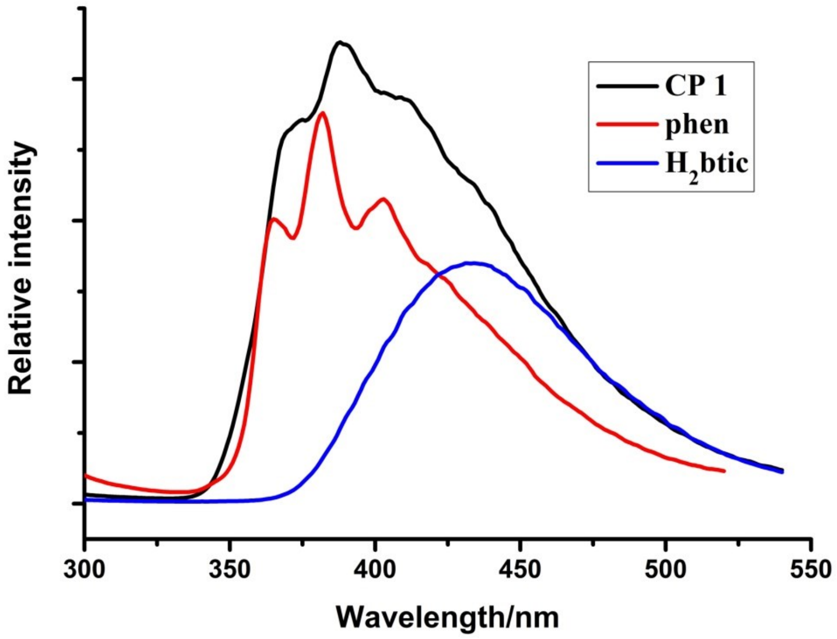

Figure S4. Solid-state photoluminescent spectra of free ligands and CP-**1** at room temperature.

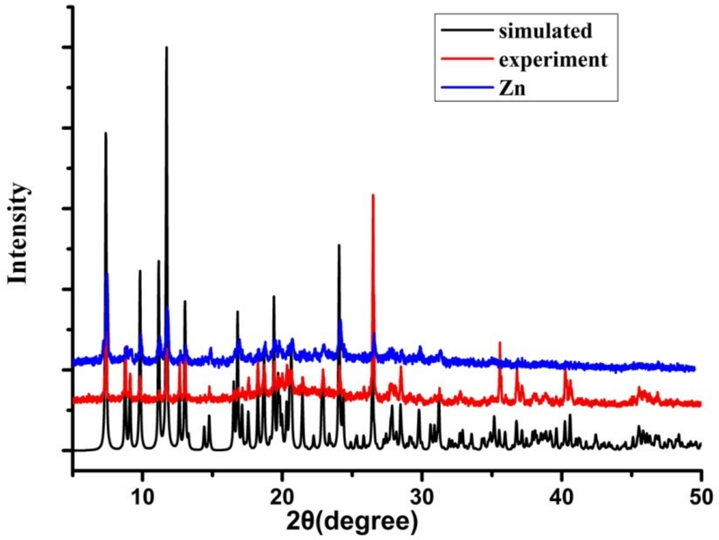

Figure S5. PXRD patterns of the simulated pattern (Black), experimental pattern (Red), and the sample of CP-**1** after Zn^{2+} test (Blue).

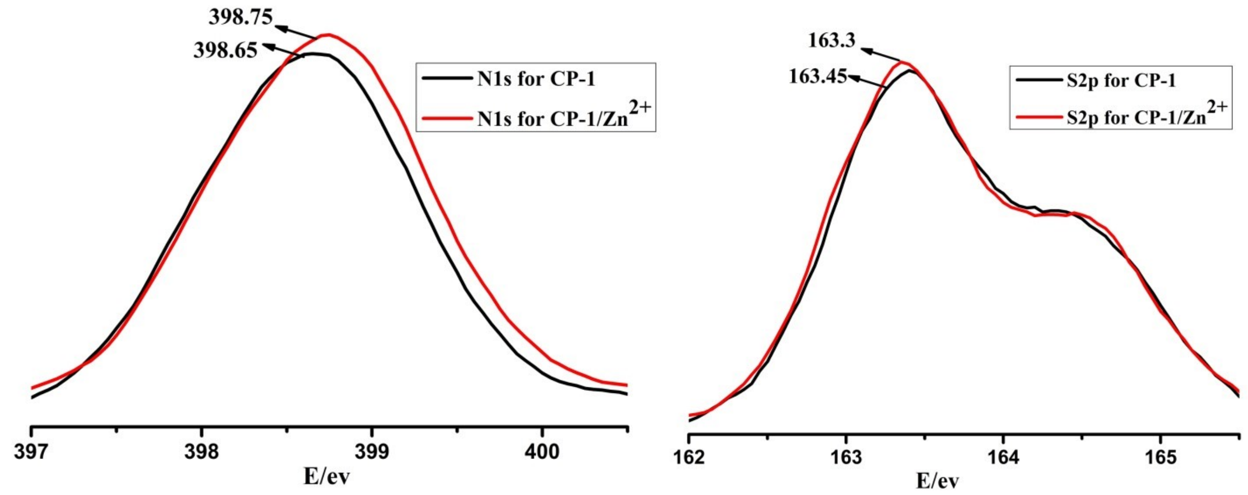

Figure S6. N 1s and S 2p XPS spectra of CP-**1** (black) and Zn^{2+}-incorporated CP-**1** (red) activated by 0.001 mol/L aqueous solution of Zn^{2+}.

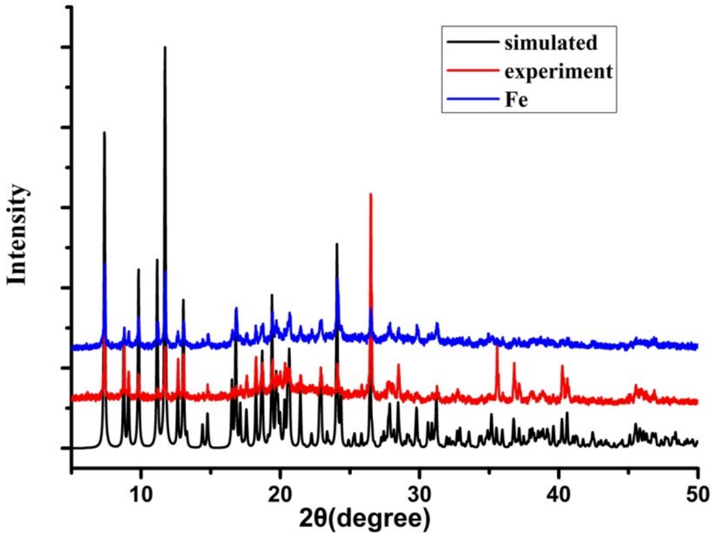

Figure S7. PXRD patterns of the simulated pattern (Black), experimental pattern (Red), and the sample of CP-**1** after Fe^{3+} test (Blue).

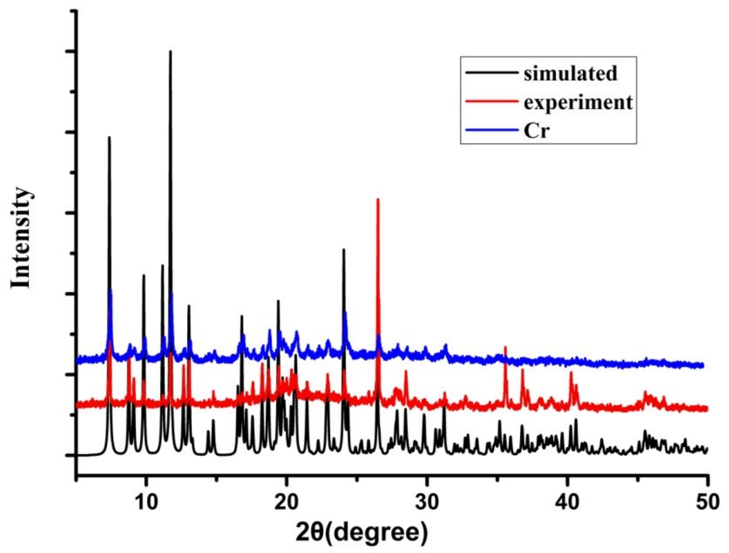

Figure S8. PXRD patterns of the simulated pattern (Black), experimental pattern (Red), and the sample of CP-**1** after $Cr_2O_7^{2-}$ test (Blue).

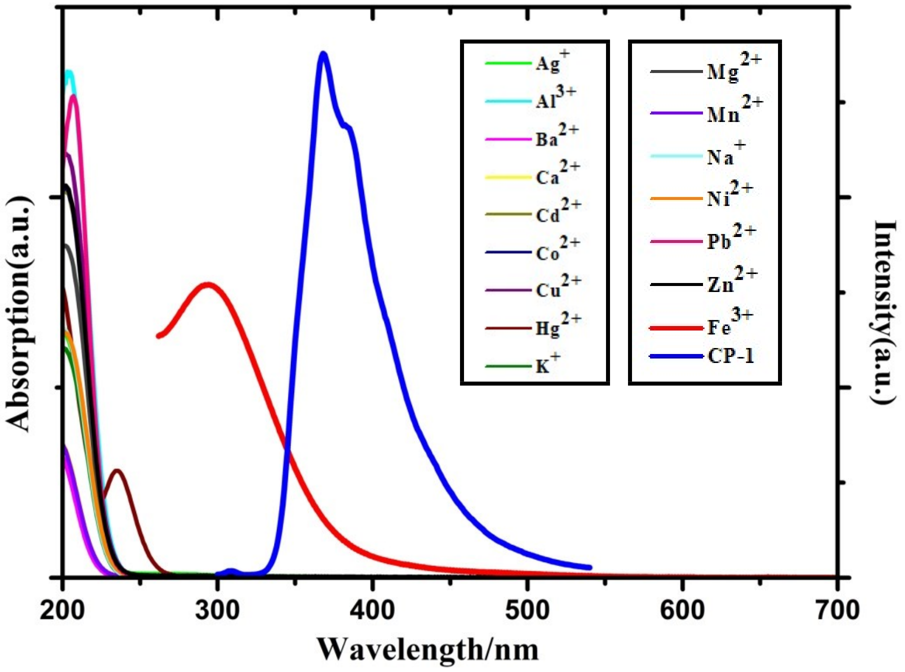

Figure S9. UV-vis

absorption spectra of varied metal ions and the emission spectra of CP-**1** in water.

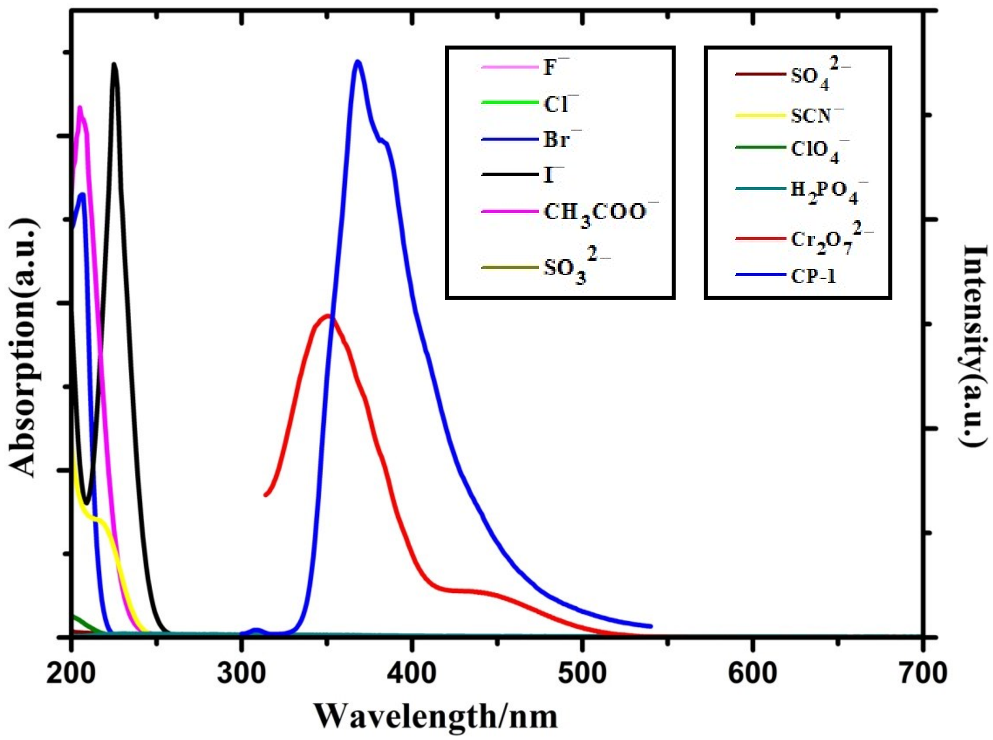

Figure

S10.

UV-vis

absorption spectra of varied anions and the emission spectra of CP-**1** in water.
